# Optimisation of reduction for prolapsed silicone tube after lacrimal intubation

**DOI:** 10.1017/S0022215123001706

**Published:** 2024-05

**Authors:** Xiao Shen, Chunlian Huang, Shili Wang, Jing Wen

**Affiliations:** Department of Ophthalmology, Taizhou Central Hospital (Taizhou University Hospital), Taizhou, China

**Keywords:** Infiltration anesthesia, lacrimal duct obstruction, dacryocystorhinostomy

## Abstract

**Background:**

A common complication of bicanalicular intubation is dislocation of the silicone tube.

**Methods:**

Eleven patients with prolapsed silicone tubes who had undergone bicanalicular nasal intubation were injected with a 2 per cent lidocaine solution to infiltrate the lacrimal duct mucosa. A memory wire probe was used to pull a 4-0 suture through the lacrimal passage retrogradely, allowing the suture to grab the silicone tube. Paraffin oil was applied to the contact part of the rope and the silicone tube, then the distal end of the silk thread was removed from the nostril until the tube was pulled into place.

**Results:**

The prolapsed silicone tubes were restored by surgery in nine patients, with the drainage tube in the correct position in the eye and the lacrimal duct irrigation unobstructed.

**Conclusion:**

The optimisations made in this study are considered effective adjustments of reduction surgery for a prolapsed silicone tube.

## Introduction

Lacrimal duct obstruction is one of the most common eye diseases and can be congenital or acquired. Congenital obstruction may occur anytime during fetal development and spontaneous canalisation may happen between 6 and 12 months of age.^1^ In the case of acquired lacrimal duct obstruction, the causes are complex and may include complications after eye surgery or dacryoliths.

Epiphora is the main symptom of blocked nasolacrimal ducts and the accumulation of tears in the eye can lead to excessive tearing and even infection.^2,3^ A previous study showed that 40.7 per cent of patients with epiphora had an obstruction in the lower portion of the lacrimal system and 8 per cent had an obstruction in the upper portion of the nasolacrimal system.^4^ Earlier research revealed that 31.8 per cent of patients had nasolacrimal duct obstruction.^5^ More importantly, both acquired and congenital obstruction of the nasolacrimal duct can result in tear stasis in the lacrimal sac.^6^ If the blocking is not treated promptly, lifelong epiphora may result, which can seriously affect the patient's quality of life.^7^ Thus, the treatment of lacrimal duct obstruction has received increasing attention in recent years.

Common treatments for lacrimal duct obstruction include medication and surgery. Bicanalicular intubation has been widely used to treat punctum stenosis and obstruction, as well as traumatic lacerations of the lacrimal drainage system.^8^ This surgery is easy to perform, does not affect appearance and can achieve relatively satisfactory curative effects. Moreover, Ritleng bicanalicular silicone intubation is an effective technique for the short- and long-term treatment of congenital nasolacrimal duct obstruction.

In addition, in an earlier retrospective study, bicanalicular silicone intubation therapy was used with good results to treat lacrimal duct stenosis and obstruction in patients with allergic conjunctivitis. However, the success rate of intubation surgery may be affected by numerous factors, such as the age of patients, the degree of nasolacrimal duct stenosis and the pre-operative condition of the lacrimal duct. All these factors may lead to diverse complications, with dislocation of the silicone tube being one of the most frequent.

There are many causes of silicone tube dislocation, including a thick knot at the distal end of the drainage tube, an inappropriate length of silicone tube and improper post-operative maintenance of the patient.^9^ Importantly, the dislocation can easily lead to lacrimal duct obstruction and adhesion, as well as a risk of irritation or distress to patients and infection, which affects the therapeutic outcome and the patient's quality of life after surgery.^10^ It is therefore vital for patients to undergo a timely reduction of the silicone tube length.

In this study, a new anaesthetic method and surgical material were used to ameliorate the reduction surgery, which in turn improved the treatment outcome and reduced the patient's pain during the repositioning process.

## Materials and methods

### Patients

A total of 11 patients who underwent bicanalicular silicone tube intubation between March 2020 and December 2021 were enrolled in the present study, with 5 cases of lacrimal duct obstruction, 2 cases of chronic lacrimal duct inflammation, 3 cases of canalicular laceration and 1 case of lacrimal canaliculus laceration.

**Figure 1.**
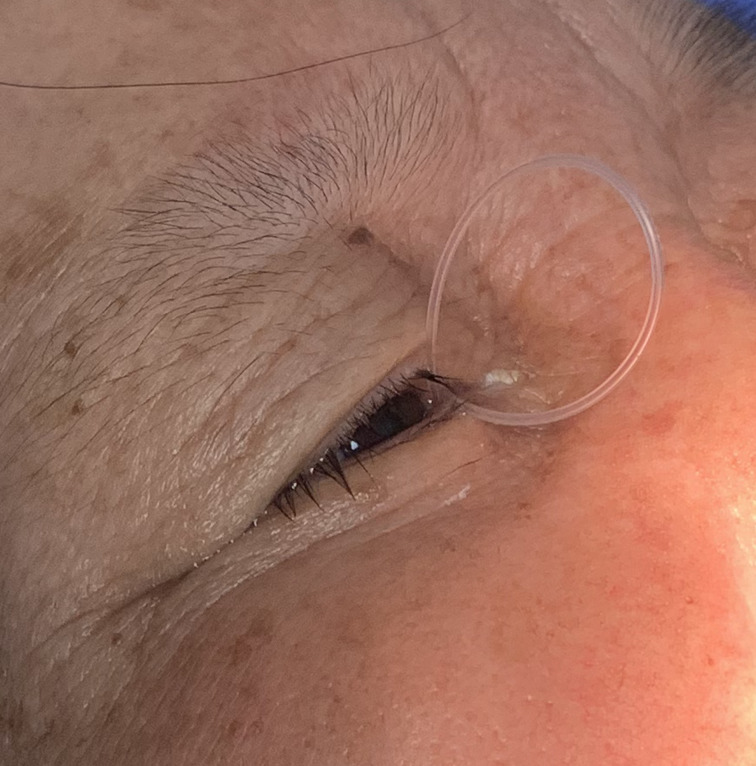
Patient with prolapsed silicone tubes. The silicone tube can be seen slipping out of the medial canthus.

**Figure 2.**
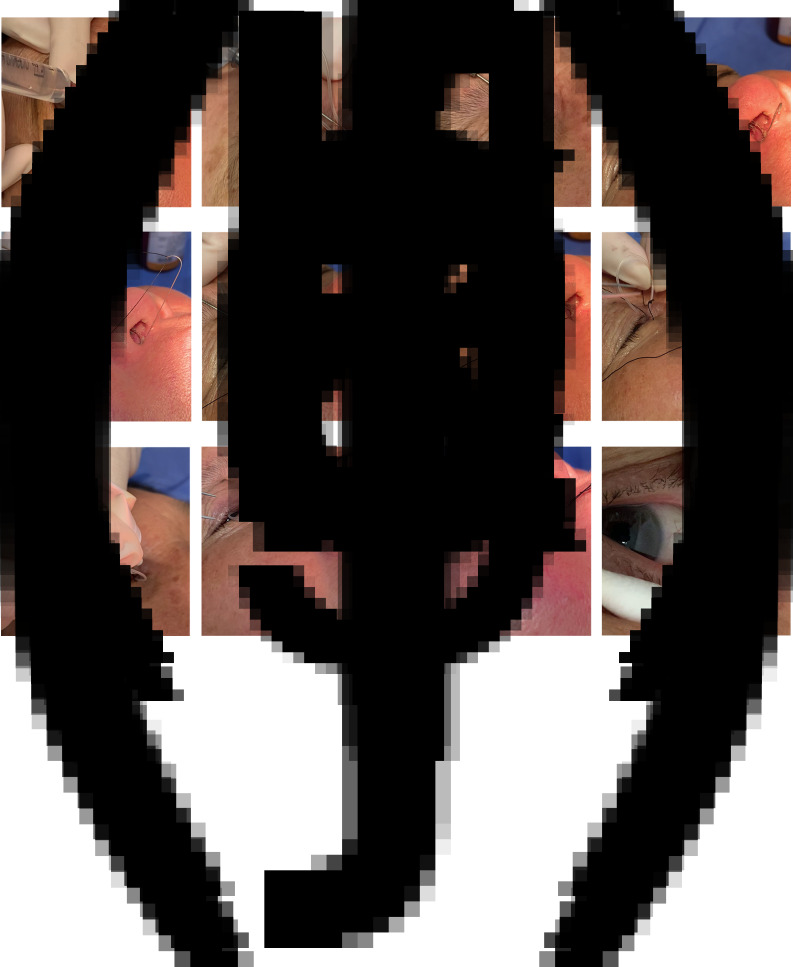
Repositioning tubes: (a) topical anaesthesia with 0.5 per cent proparacaine eye drops; (b) subcutaneous infiltration anaesthesia with an injection of 2 per cent lidocaine solution; (c) the memory wire probe is inserted into the lacrimal canaliculus, travels parallel to the eyelid margin, touches the bone wall of the lacrimal sac and then turns downward and protrudes down the nasolacrimal duct into the inferior meatus; (d) the probe is turned so that the head of the memory wire can stick out of the nasal cavity; (e) a 4-0 silk thread is pulled through the head of the memory wire; (f) the memory guide wire is pulled so that the probe, together with the suture, is pulled back into the lacrimal duct retrograde until it leaves the punctum; (g) the suture is separated from the memory guide wire; (h) one end of the 4-0 silk thread is pulled out of the puncta, wrapped around the silicone tube and tied into a ‘noose’; (i) paraffin oil is applied to the contact part of the rope and the silicone tube; (j) the silicone tube ring is secured with one hand and the distal end of the silk thread is pulled out of the nostril with the other hand; (k) the silicone tube is pulled into place through the silk thread and can be seen pulled out from the nostril; (l) the position of the silicone tube is correct after repositioning.

The inclusion criteria were as follows: (1) patients with silicone tube dislocation after lacrimal intubation surgery (Figure 1); (2) patients whose silicone tube dislocation occurred within three months after the intubation; and (3) patients compatible with local anaesthesia. The exclusion criteria were as follows: (1) patients whose silicone tube had been dislocated for more than three months from the time of surgery; (2) patients with active bleeding in the nasal cavity or infectious inflammation of the tear duct; and (3) patients with incomplete or broken silicone tubes.

All the surgical procedures were performed under local anaesthesia. The study was approved by the Ethics Committee of Taizhou Central Hospital (Taizhou University Hospital) (2022L-08-05). All methods were performed in accordance with the relevant guidelines and provisions of the Declaration of Helsinki, and all subjects and/or their legal guardians have signed an informed consent form.

### Probe with memory guide wire

**Figure 3.**
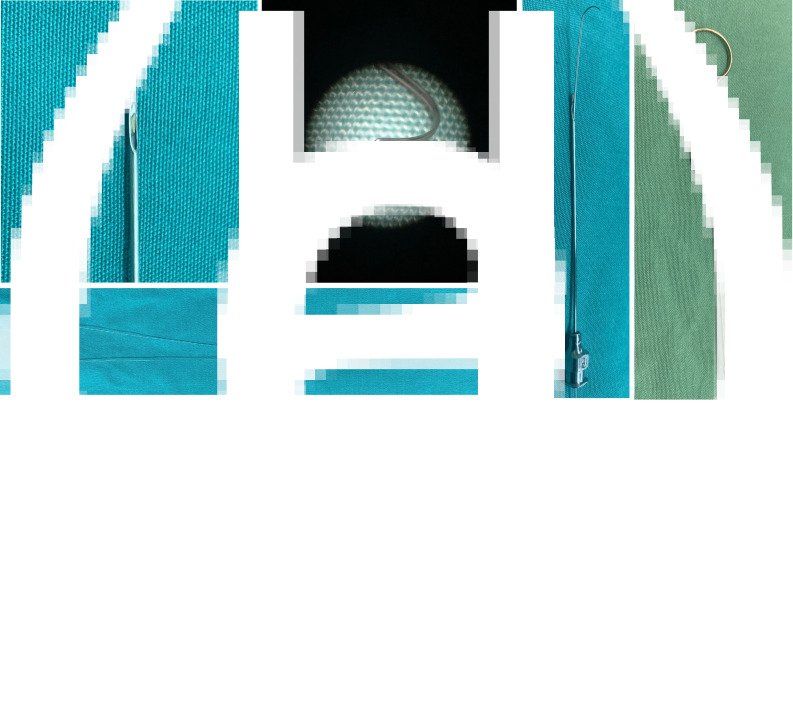
The memory wire probe used in this surgery. A hollow stainless-steel probe (a) with an internal memory wire (b, c) constitutes the memory wire probe (d). The probe has a closed circular tip and a side port that allows the memory wire to enter and exit. The memory wire is made of a single piece of memory metal wire bent into two strands, capable of grasping threads. The metal hook (e) is used to hook the memory wire inside the nasal cavity.

A probe with a memory guide wire was used to reposition the dislocated drain (Jinan Runshi Medical Equipment Co., Ltd). The probe has a closed, rounded tip and a side port that allows the memory guide wire to enter or exit. The orientation of the port is the same as the number ‘9’ engraved on the syringe adapter (Figure 3). The memory guide wire is made of titanium-nickel alloy and is folded into the shape of an elbow and a straight body (Figure 3C), which conforms to the characteristics of the inferior nasal meatus. When the tail of the memory guide wire is pushed, the head protrudes from the nostril along the inferior nasal meatus and the softness of the guide wire reduces the damage to the nasal mucosa. Furthermore, the wire can recover from deformation, allowing it to grip the silk thread and pull it back into the probe.

Retractor for hooking the memory guide wire has a gourd-shaped tail to make it easy to hold. The hooked head facilitates pulling the memory guide wire into the inferior nasal meatus.

### Reposition process

The tubes were repositioned as follows (Figure 2). Firstly, for local anaesthesia, 0.5 per cent proparacaine eye drops (Alcaine; Alcon-Couvreur SA, Belgium) were applied three times to the affected eye. The lacrimal duct irrigation probe was then inserted into the lacrimal sac through the upper punctum, a 2 per cent lidocaine solution was injected to infiltrate the lacrimal duct mucosa and subcutaneous infiltration anaesthesia was performed on the eyelids near the upper and lower lacrimal canaliculus.

Next, the memory wire probe was gently inserted into the lacrimal canaliculus via the superior lacrimal punctum and moved parallel to the eyelid margin. When the lacrimal sac bone wall was touched, the probe was moved down along the lower edge of the nasolacrimal duct and into the inferior meatus. The operation process must be gentle to avoid the formation of false passages.

The probe was turned so that the side marked with a ‘9’ faced the tip of the nose. The tail of the memory guide wire was then gently pushed to allow its head to pass out of the probe. In most cases, the head of the memory guide wire stuck out of the nasal cavity. If the coiled guide wire could not do this by itself, it could be pulled out with a retractor deep into the inferior meatus.

A 4-0 suture thread was then taken through the head of the memory wire and the head was retracted into the probe by pulling the memory guide wire. The probe was pulled back into the lacrimal duct retrogradely, together with the suture, until it left the punctum. Finally, the memory guide wire was separated from the silk thread.

One end of the 4-0 suture thread was pulled out of the punctum, wrapped around the silicone tube and tied into a ‘noose’ to grab the silicone tube. Paraffin oil was applied to the contact part of the rope and the silicone tube to allow the silicone tube to slide easily into the lacrimal duct and reduce the cutting effect of the silk thread on the tissue.

The silicone tube ring was held in place by the surgeon with one hand, while the distal end of the silk thread was carefully pulled out of the nostril with the other hand. The ‘noose’ then covered the silicone tube from the upper punctum and slid into the lacrimal canaliculus to the lacrimal sac until it was stopped by the knot of the silicone tube.

The distal end of the silk thread was held with traction until the silicone tube was pulled into place. The silicone tube could be seen in the nostril, with the knot of the drainage tube visible. Finally, the silk coil was cut. The repositioned drainage tube could be seen in the medial canthus.

## Results

A total of 11 cases (5 males and 6 females) with a mean age of 48.9 ± 12.8 years (range, 24–66 years) were enrolled in this study. The median number of days between the onset of dislocation symptoms and the time to surgery was 10 days, with an interquartile range of 7 to 14 days. Among the patients, 9 had their prolapsed silicone tubes restored through surgery and 2 had failed reduction, giving a success rate of 81.82 per cent. Patients with successful reduction had their drainage tubes in the correct position, and lacrimal duct irrigation was unimpeded. For patients who had failed reduction surgery, part of the drainage tube remained in the lacrimal duct and was not removed.

The surgery was an out-patient procedure that took about 20 minutes. During the operation, the drainage tube was fixed using an absorbable suture that could be broken down after one month. Patients had no obvious pain during the operation. Patients with successful surgery were reviewed 1 day, 1 week, 1 month and 3 months after the reduction to check the position of the drainage tube and flush the lacrimal duct. In addition, they retained their silicone tubes for three months without recurrence of prolapse. Nine patients had no obstructed lacrimal passage irrigation.

## Discussion

Lateral prolapse of the silicone tube is a frequent complication of bicanalicular intubation.^11^ Depending on its severity, silicone tube dislocation can be divided into partial dislocation and complete dislocation.^8^ In partial dislocation, the silicone tube can be pushed back to the original position with tweezers to restore the same therapeutic effect as before. For complete dislocation, where the silicone tube cannot be restored to its original position using only forceps, a drainage tube reduction operation is required.

Previous research reports and reviews suggest that 17.5 per cent of patients develop complications of premature catheter dislocation.^12,13^ Furthermore, failure to reattach the tube promptly may result in complications such as canalicular or punctum damage, infections, corneal erosions, granuloma and fistula formation.^14–16^ Timely and successful repositioning of the prolapsed silicone tube is therefore very important.

There are many causes of dislocation. Improper handling by patients after surgery, such as excessively rubbing the medial canthus and pulling the silicone tube, will result in the tube moving away from the medial canthus. Previous studies indicated that prolapse and displacement may be related to the use of a single knot to fix the drainage tube.^13^ Apart from the advantages of a convenient and fast operation, single-node fixation also has flaws, such as irritation of the nasal mucosa and formation of granulomas, resulting in prolapse and displacement of the silicone tube.

The method described in this study is primarily used for the reduction of dislocated silicone tubes after lacrimal silicone tube implantation, including simple lacrimal intubation and combined endonasal dacryorhinocystostomy (DCR) with lacrimal intubation procedures. Some patients who undergo endonasal DCR surgery may have associated lacrimal duct obstruction or lacrimal tube obstruction. For these patients, simultaneous silicone tube implantation is typically performed during the DCR procedure. If dislocation occurs after surgery, this method can also be used for reduction.

Among the patients with tube prolapse involved in this study, there were two cases of failed reduction. Both cases came to our hospital for treatment after silicone tube prolapse following intubation surgery in other hospitals. During the diagnosis process, we found that the distal end of the drainage tube was knotted thickly and was difficult to pull. We believed that the failure was as a result of the thick knot being tightly embedded in the nasolacrimal duct or lacrimal sac. When the knot was pulled by the silk thread, the tension broke the silicone drainage tube, with the knot remaining in the nasolacrimal duct. We therefore used absorbable thread that disintegrates after one month for binding during the reduction and canalicular intubation procedures. This prevented problems such as movement of the drainage tube and failed reduction owing to the thick knot.

We also applied paraffin oil to the contact part of the suture and the silicone tube to allow the silicone tube to slide more smoothly into the lacrimal duct and reduce the risk of snagging. The paraffin oil also reduced the cutting friction of the silk thread and the silicone tube in the tissue, and alleviated the bleeding caused by friction during surgery.

- A common complication of bicanalicular intubation is dislocation of the silicone tube
- Failure to reattach the tube promptly may result in complications
- Patients who experienced successful reduction retained the silicone tube for three months with no further prolapse
- The optimisations described in this study can improve the surgical experience for patients and reduce the risks of the procedure

The surgical cases involved in the present study were all anaesthetised with lidocaine perfusion in the lacrimal duct. In previous studies, general anaesthesia,^17^ topical anaesthesia^13^ and no anaesthesia were commonly used.^9^ However, most types of general anaesthesia are associated with peri-operative and post-operative complications. Compared with general anaesthesia, surface anaesthesia lowers the dose of the drugs and has the advantages of being quick, simple and non-invasive. However, traditional local anaesthesia methods can only work on the surface of the eye, so the patient still experiences pain as the silicone tube moves through the lacrimal and nasolacrimal ducts during the reduction. Lidocaine instillation of the lacrimal duct can directly anaesthetise the lacrimal duct mucosa, therefore patients feel less pain and have a better surgical experience compared to that with traditional ocular surface anaesthesia.

## Conclusion

Absorbable thread was used to tie the silicone tube and carry out the lacrimal infusion method for anaesthesia in prolapsed silicone tube reduction surgery to reduce the risk of surgical failure, silicone tube breakage and entrapment in the nasolacrimal duct as a result of the procedure itself.

Silicone tube reduction surgery takes about 20 minutes and can be done in an out-patient clinic without hospitalisation. Replacing secondary nasal cannula with reduction surgery can reduce the overall operation cost and mitigate psychological stress and secondary injuries on the patient. In addition, under subcutaneous infiltration anaesthesia, patients should feel less pain during the surgery. These optimisations can improve the surgical experience for patients and reduce the risks of surgery, thus providing new options for the surgical repositioning of lacrimal duct silicone tubes.

## Data Availability

The image data used to support the results of this study are included in the article.
